# Exploring the Relationships between Gas Dispersion Parameters and Differential Pressure Fluctuations in a Column Flotation

**DOI:** 10.1021/acsomega.1c01955

**Published:** 2021-08-16

**Authors:** Xiangning Bu, Shaoqi Zhou, Meng Sun, Muidh Alheshibri, Md. Shakhaoath Khan, Guangyuan Xie, Saeed Chehreh Chelgani

**Affiliations:** † Key Laboratory of Coal Processing and Efficient Utilization of Ministry of Education, School of Chemical Engineering and Technology, China University of Mining and Technology, Xuzhou, Jiangsu 221116, China; ‡ Fengxian Power Supply Co., Ltd., State Grid Jiangsu Electric Power Co., Ltd., Fengxian, Jiangsu 221700, China; § Department of Basic Science, Deanship of Preparatory Year and Supporting Studies, Imam Abdulrahman Bin Faisal University, P.O. Box 1982, Dammam 31441, Saudi Arabia; ∥ Basic & Applied Scientific Research Center, Imam Abdulrahman Bin Faisal University, P.O. Box 1982, Dammam 31441, Saudi Arabia; ⊥ ARC Research Hub for Computational Particle Technology, Department of Chemical Engineering, Monash University, Clayton, VIC 3800, Australia; # Minerals and Metallurgical Engineering, Department of Civil, Environmental and Natural Resources Engineering, Luleå University of Technology, SE-971 87 Luleå, Sweden

## Abstract

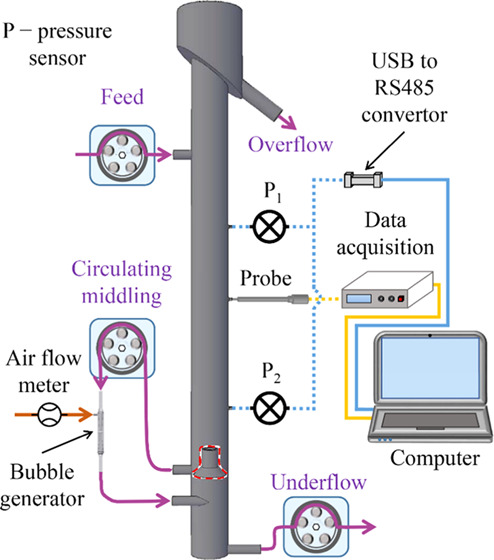

Flotation separation, which is the most important mineral beneficiation technique, is dependent on gas dispersion (hydrodynamic conditions). Thus, many investigations have focused on the precise determination of hydrodynamic conditions such as Reynolds number of the bubbles, bubble velocity, and bubble diameter. However, few studies have examined their relationships with pressure fluctuations in a column flotation. This study introduced the differential pressure fluctuations as an actual variable that could be considered to determine the collection zone’s hydrodynamic conditions in a cyclonic microbubble flotation column. In general, the outcomes indicated that superficial gas velocity had the most substantial relationship with the differential pressure fluctuations among other flotation factors (such as pump speed, superficial gas velocity, superficial water velocity, and frother dosage). Furthermore, a high coefficient of determination (*R*^2^ > 0.77) for the equation generated to assess the relationships demonstrated that differential pressure fluctuations could be used as a promising tool to determine the hydrodynamic parameters’ characteristics in the flotation columns.

## 1 Introduction

Froth flotation is the most important separation technique among the mineral beneficiation methods. Several subprocesses, including bubble–particle collisions, attachment, and detachment, are involved in flotation separation.^1^ Hydrodynamic variables such as bubble size, gas velocity, and Reynolds number of the bubbles, which are the bubble (gas) dispersion parameters, play an essential role in collecting hydrophobic particles in the pulp zone.^1,2^ Therefore, their determination is important, and different methods have been developed to measure some of them, notably the bubble size, gas velocity, gas holdup, and bubble surface area.^3^ Mechanical flotation machines have dominated in the mineral-processing industries worldwide since the early days of froth flotation, while flotation columns with a thick froth layer afford superior performance in terms of nonselective entrainment inhibition.^4−8^ It was documented that the bubble surface area flux has a direct relationship with the recovery of hydrophobic particles (true flotation), which is affected by the superficial gas velocity and bubble size distribution in column flotation.^3,9,10^ It is reported that gas holdup is correlated with bubble surface flux.^10,11^ Unlike the bubble surface flux, the easy-to-measure gas holdup can be used in the control and optimization of industrial flotation machines.^12,13^

**Figure 1:**
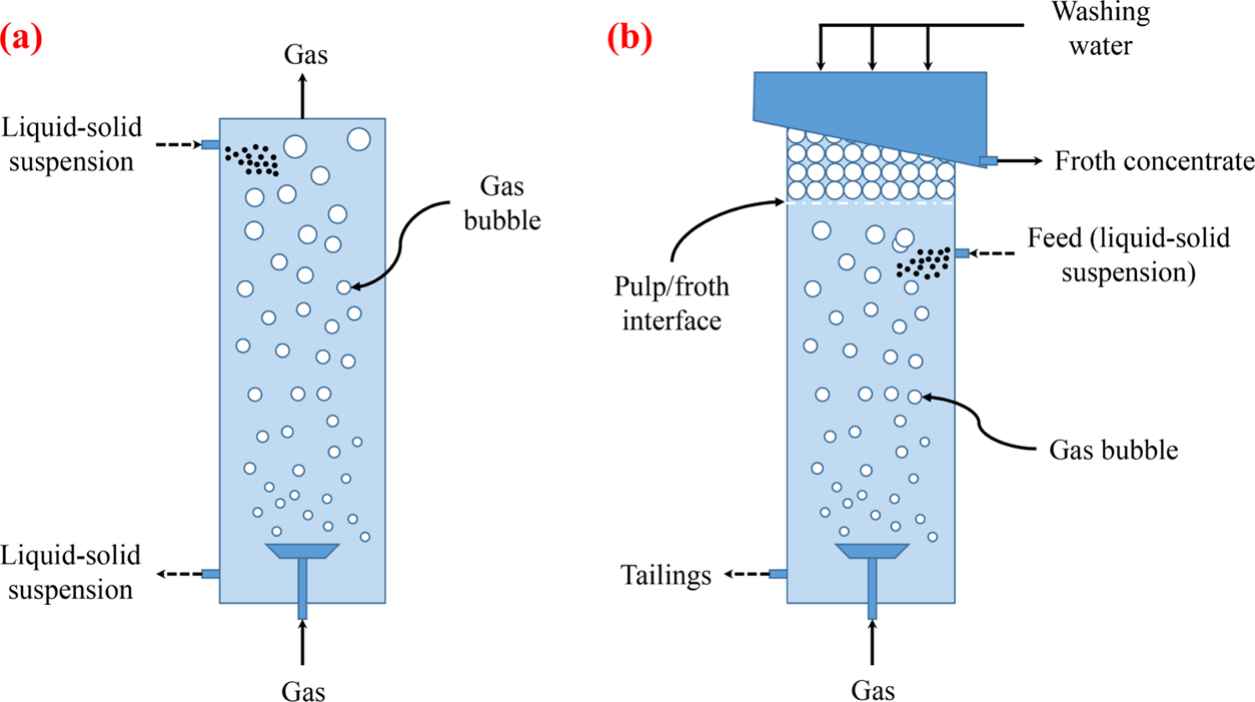
Schematic diagram of the similarities between (a) a gas–liquid–solid bubble column reactor and (b) a flotation column.

For mineral flotation, bubble size distribution in two- and three-phase flotation devices can be measured using visual measuring techniques combined with range-processing software.^2,3,9^ Bubble size in the pulp zone is commonly measured by placing a vertical pipe into the measurement point. The two typical technology schemes are the McGill University bubble sizing method (imaging)^14^ and the University of Cape Town (UCT) device (capillary).^15^ Maldonado and Gomez^12^ have found that the conductivity-based gas holdup sensor has been widely used at the lab and industrial scale for diagnostic purposes compared to other measurement methods of collection-zone gas holdup in flotation cells. As illustrated in Figure 1, flotation columns can be considered a specific application of gas–liquid–solid bubble column reactors in the mineral process from the multiphase flow perspective. Thus, the measurement methods used for hydrodynamics studies in flotation columns can be covered by those of bubble column reactors. Boyer et al.^16^ conducted a systematic review of hydrodynamic parameters’ measurement technology in gas–liquid and gas–liquid–solid multiphase flow reactors. They divided the hydrodynamic parameter measurement methods into noninvasive and invasive methods according to the measurement principle.

Noninvasive measurement methods mainly include wall pressure fluctuations, dynamic gas separation technology, visualization technology (photography/video technology, ray technology, particle image velocimetry technology, and nuclear magnetic resonance technology), laser Doppler and its derivative technology, radioactive particle tracing technology, and tomography technology (γ/X-ray, capacitance/resistance, or ultrasonic technology), etc.^12^ Camera and image-processing technology is the main technology of bubble feature measurement. Its limitations are mainly reflected in the difficulty of obtaining high-quality images and efficient image analysis software. The ghosting of bubbles in three-dimensional (3D) equipment and the deterioration of image resolution under high-solid-content conditions are the bottlenecks of applying this technology. Particle image velocimetry technology, laser Doppler technology, and radioactive particle tracking technology are the main methods for measuring the velocity of bubbles. The common feature is the high application cost, which is mainly suitable for gas–liquid two-phase systems. Radioactive particle tracking technology can be used to observe the gas–liquid–solid three-phase system, but it has a greater safety challenge because this technology uses radioactive materials. At the same time, the acquisition and use of radioactive particles require the approval of relevant national authorities and involve huge costs. Tomography technology is suitable for monitoring phase holdup in the gas–liquid two-phase system and gas–liquid–solid three-phase system. The high cost and the reconstruction algorithm reliability are factors that need to be considered in the application process. Both dynamic gas separation technology and pressure fluctuation technology analyze the flow pattern, phase holdup, and bubble size by monitoring the changes in the wall pressure of the multiphase flow reactor. This technology has a low cost and can be used in industrially complex multiphase flow systems. The application of noninvasive technologies such as optical technology and tomography technology in the research of bubble flow has made great progress. Compared with noninvasive technology, the advantages of invasive measurement technology could not be ignored in high-turbulence systems. Invasive measurement technology can be divided into needle probe measurement technology, heat transfer probe measurement technology, ultrasonic probe measurement technology, and pitot tube measurement technology according to the different test probes.^16^

**Figure 2:**
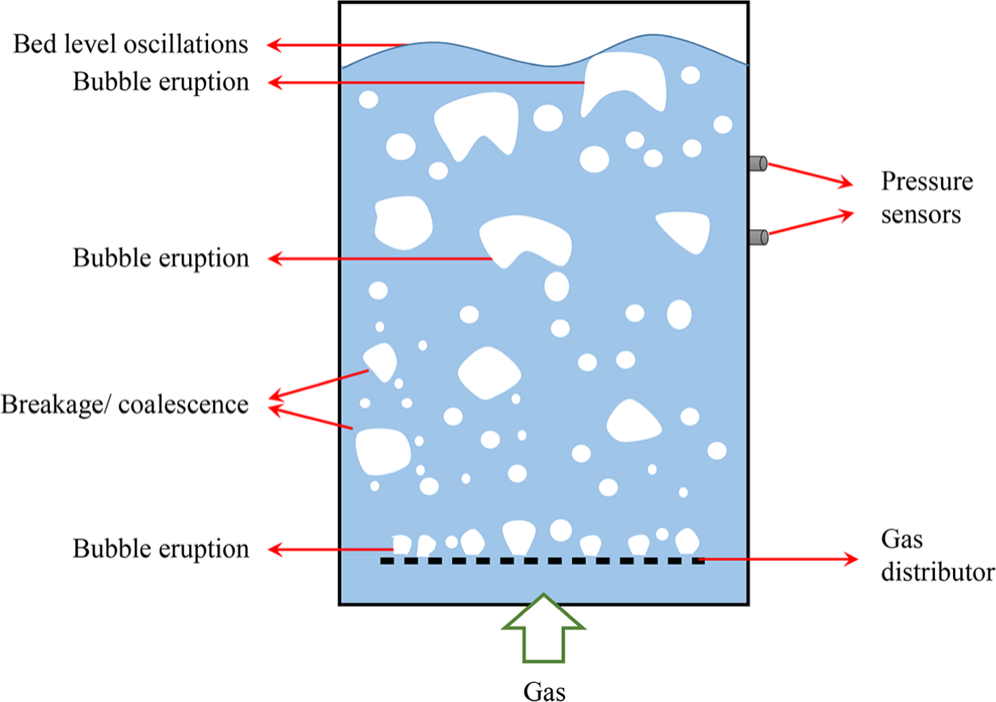
Schematic representation of the sources of the pressure fluctuations in gas–liquid flow (adapted in part from Letzel et al.^17^).

Notably, a method based on pressure fluctuations has been widely used to characterize the hydrodynamics of bubble columns and fluidized beds, which can be easily measured even under harsh industrial conditions.^17,18^ This technology has a low cost and good stability under industrially complex production conditions.^17−20^ The pressure-measuring system includes a pressure sensor and a pressure-measuring nozzle, which is sturdy and durable, relatively cheap, almost noninvasive, and avoids the disturbance of the fluid at the measuring point.^3,12^ The fluctuation of the pressure signal is mainly related to the movement of the bubbles inside the bed, but the exact source of the fluctuation is still controversial.^21^Figure 2 shows a schematic representation of the sources of pressure fluctuations in a bubble column. First, there occurs the bubble formation and detachment from the distributor. As the bubbles rise through the column, they continuously coalesce and break up, again causing pressure fluctuations. Also, when a bubble erupts at the surface, a pressure fluctuation results. Another source for fluctuations is the wake of the bubbles. The wake oscillates, making every bubble a transmitter of pressure fluctuations. Furthermore, the overall pressure in the wake is lower than in the rest of the column. This pressure trough is observed when the bubbles pass the pressure probes nearby. Bed-level oscillations and macro circulations also cause pressure fluctuations. Pressure fluctuations are mainly related to bubble motion within multiphase systems, providing more comprehensive information on the hydrodynamics of a bubble column reactor (or a fluidized bed).^17,18,22^ The sources of the pressure fluctuations in gas–liquid flow include bubble formation, breakup and coalescence, bubble eruption at the liquid surface, the wake of bubbles, liquid-level oscillations, and macro-flow circulation.^17^ For the characterization of gas–solid fluidized beds’ dynamics, time-series analysis of pressure fluctuations can be performed in the time domain, frequency domain, and state space. Compared to the frequency domain and state-space analysis, time-domain analysis is the most straightforward approach for identifying regimes in fluidized beds and bubble columns.^18,23−25^ However, the frequency domain and state-space analysis can provide better prediction performance for the characterization of flow regimes compared to statistical analysis.^18^

In this study, a primary exploration of the gas dispersion prediction of the collection zone in a column flotation was performed based on a statistical analysis of pressure fluctuations. Therefore, it is conceivable that pressure fluctuations can be considered an alternative approach for assessing gas dispersion in a flotation column. For the first time, this article is going to use time-domain analysis and primarily assess the relationship between flotation operating variables (height, superficial gas and water velocity, and frother dosage) and the pressure fluctuations. On the other hand, the potential of using pressure fluctuations for assessing the gas dispersion of the collection zone was examined in a cyclonic microbubble flotation column.

## 2 Results and Discussion

### 2.1 Pressure Fluctuations

#### 2.1.1 Height

**Figure 3:**
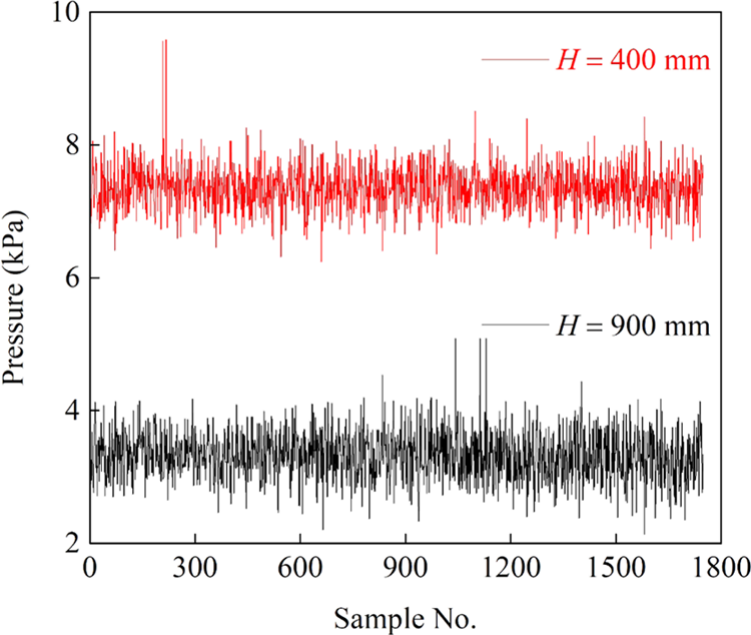
Time series of the pressure signals at heights of 400 and 900 mm. The height at the bottom of the flotation column was set as 0 mm (*n* = 500 rpm, *C*_f_ = 0.15 mmol/L frother, *J*_w_ = 0.043 cm/s).

**Figure 4:**
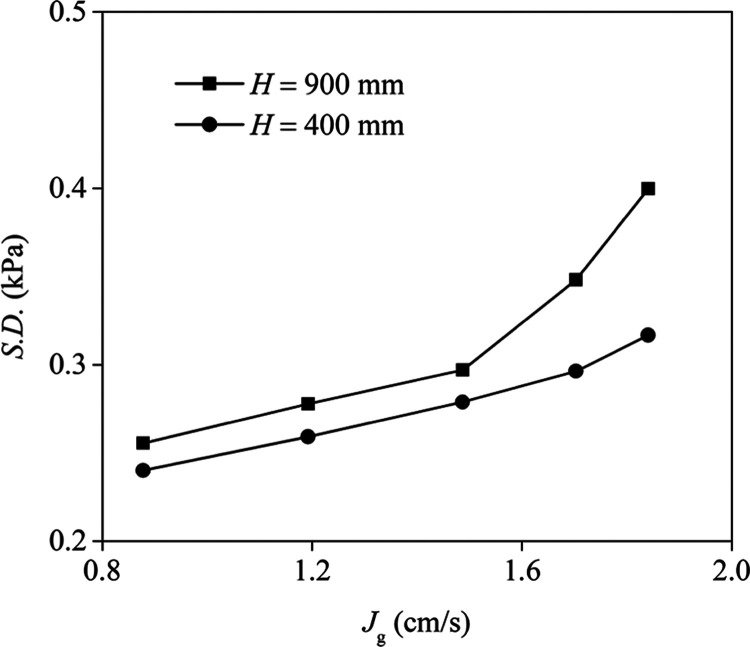
Calculated S.D. values of pressure fluctuations at heights of 400 and 900 mm (*n* = 500 rpm, *C*_f_ = 0.15 mmol/L frother, *J*_w_ = 0.043 cm/s).

As it was predictable, the pressure is higher at the lower point (Figure 3). The calculated standard deviation (S.D.) values of pressure fluctuations at heights of 400 and 900 mm under various superficial gas velocities showed that the pressure fluctuation amplitude increased with an increasing height of the measurement point (Figure 4). This phenomenon may be due to bubble coalescence and changes in bubble size. Chilekar et al.^26^ found that bubbles’ passing results in a pressure drop at the pressure sensor’s measurement position.^12^ Meanwhile, the amplitude of the pressure fluctuations exhibits an increasing trend as the height of the measurement points above the sparger increases. Moreover, Darton^27^ reported that bubbles’ coalescence leads to bubble size growth, increasing the distance above the distributor in fluidized beds.^28^ They proposed an empirical equation to describe the exponential relationship between the bubble diameter and the measurement point, demonstrating these outcomes’ consistency with previous observations.^26,27^

#### 2.1.2 Superficial Gas Velocity

**Figure 5:**
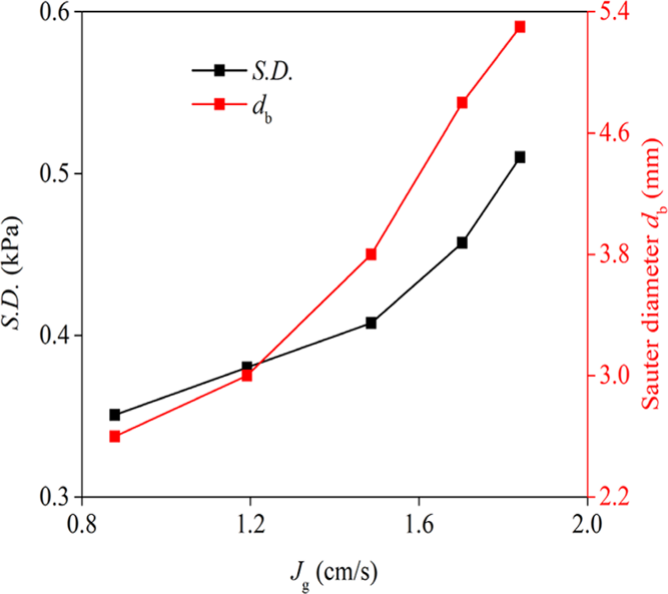
Effect of the superficial gas velocity (*J*_g_) on the S.D. of differential pressure fluctuations and Sauter bubble diameter (*n* = 500 rpm, *C*_f_ = 0.15 mmol/L frother, *J*_w_ = 0.043 cm/s).

Assessing the effect of the superficial gas velocity (*J*_g_) on the S.D. of pressure fluctuations and Sauter bubble diameter of the collection zone indicated that with an increase in *J*_g_, the average bubble size increased from approximately 3.5 to 5.3 mm (Figure 5). These increases revealed that the bubbles coalesced significantly when the *J*_g_ increased. The increase of bubble size in the pulp zone on increasing the gas velocity was reported in other investigations.^29,28,30^ During the passing of large bubbles, a sharp pressure drop occurs at the pressure measurement point. A higher rising velocity of larger bubbles leads to increased fluctuations in the liquid velocity and the bubble vicinity. In other words, the number of bubbles’ wakes increases and their size grows.^31^ The formation of large bubbles meant that the probability of bubble coalescence increased significantly, which is consistent with the average bubble diameter measurements (Figure 5) and is illustrated in another investigation.^7^ These outcomes also showed that a higher *J*_g_ increased the S.D. of differential pressure fluctuations (Figure 5). This phenomenon can be explained based on the bubble size variations and bubble rising velocity.^32^ The pressure fluctuations resulting from the coalescence and breakup of bubbles and bubble eruption on the liquid surface increases with an increase in superficial gas velocity.^17^ These phenomena could explain the increasing S.D. values of differential pressure fluctuations when the *J*_g_ was increased.

#### 2.1.3 Superficial Water Velocity

**Figure 6:**
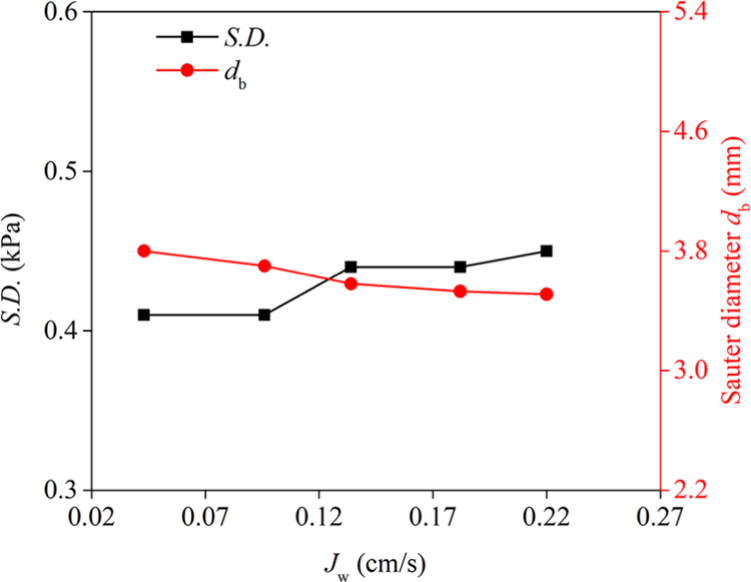
Effect of superficial water velocity (*J*_w_) on the S.D. of differential pressure fluctuations and Sauter bubble diameter (*n* = 500 rpm, *C*_f_ = 0.15 mmol/L frother dosage, *J*_g_ = 1.44 cm/s).

Exploring the effect of the superficial water velocity (*J*_w_) on the S.D. of differential pressure fluctuations and Sauter bubble diameter of the collection zone indicated that with an increase in *J*_w_, the average bubble diameter exhibited a decreasing trend (Figure 6). Vazirizadeh et al.^33^ also reported that an upward liquid velocity of 40.4 cm/s could decrease the size and number of large bubbles than the velocity of 10.1 cm/s. Notably, the variation of *J*_w_ is significantly smaller than that reported by Vazirizadeh et al.^33^ In common, a small decrease in bubble diameter occurred since a high *J*_w_ inhibits bubble coalescence. The *J*_w_ is equal to the water velocity of the overflow (upward liquid velocity). Thus, an increasing *J*_w_ decreased the bubble’s residence time, which also decreased the bubble contact probability and inhabited the bubble coalescence. The increasing *J*_w_ led to a slight increase in the S.D. of differential pressure fluctuations (from 0.41 to 0.45 kPa) (Figure 6). This slight change in S.D. indicated that *J*_w_ had an insignificant influence on the S.D. of differential pressure fluctuations compared to *J*_g_.

#### 2.1.4 Frother Dosage

**Figure 7:**
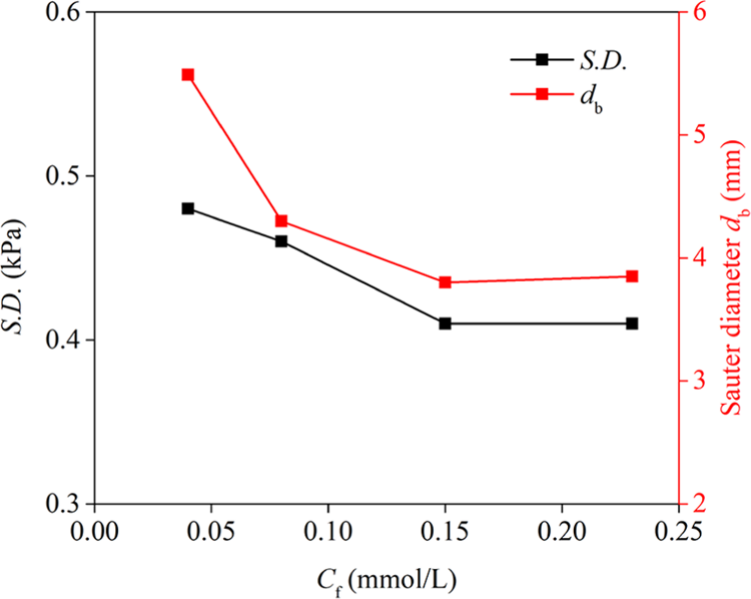
Effect of the frother dosage (*C*_f_) on the S.D. of differential pressure fluctuations and Sauter bubble diameter (*n* = 500 rpm, *J*_w_ = 0.043 cm/s, *J*_g_ = 1.44 cm/s).

Investigating the effect of the frother dosage (*C*_f_) on the S.D. of differential pressure fluctuations and Sauter bubble diameter of the collection zone (*n* = 500 rpm, *J*_w_ = 0.043 cm/s, *J*_g_ = 1.44 cm/s) showed that an increase in frother dosage significantly decreased the average bubble diameter. Similar results have also been reported in the published literature.^29,28,34^ The frother can decrease the surface tension at the liquid/gas interface, promoting an increase in bubble concentration and decrease in bubble coalescence.^34^ These outcomes (Figure 7) showed a critical coalescence concentration for *sec*-octyl alcohol of approximately 0.15 mmol/L, which is consistent with the observation of Deng et al.^35^ Therefore, the S.D. of differential pressure fluctuations decreased with an increasing frother dosage.

#### 2.1.5 Pump Speed

As *n* increased (<450 rpm), the size of the bubbles decreased significantly, which indicates that the number of large bubbles decreased at the higher pump speed of the collection zone (i.e., throat velocity of flow) (Figure 9). Fujiwara et al.^36^ observed that bubbles with a diameter of several hundred μm to about 2 mm were generated in a venturi tube. Bubbles deformed significantly because of the jet and gradually broke into pieces during flowing down due to the shear flow in the diverging nozzle. Huang et al.^37^ further demonstrated that shear stress, shed-off, and turbulence collision were all found to play a role in the bubble breakup. Thus, increasing the pump speed significantly promotes the interaction between the bubbles and turbulent flow, leading to the large bubbles breaking into several small bubbles gradually. With the disappearance of large bubbles and the generation of small bubbles, the size distribution of bubbles becomes homogeneous, which is conducive to the decrease in the S.D. of differential pressure fluctuations. However, as *n* further increased (greater than 450 rpm), the average number of bubbles decreased, while the S.D. of differential pressure fluctuations began to increase. This phenomenon can be due to the formation of an air column in the cyclone zone, resulting in severe turbulence through the axial column.^7,38^

#### 2.1.6 Correlation Assessments

Pearson correlation was used to assess the relationships between the examined variables and the S.D. of differential pressure fluctuations of the collection zone. Pearson correlation (*r*) measures the linear relationships among all variables and ranges from −1 to +1. The *r*-value sign displays the correlation direction, and its absolute value demonstrates the strength (larger absolute values indicating more substantial interdependence). Generally, the absolute *r* being higher than 0.6 indicates a strong relationship between the two variables. The outcomes (Table 1) were consistent with the above sections’ concluded results that there is a negative correlation between S.D. and *C*_f_, *J*_w_, and *n*. On the other hand, *J*_g_ and *n* had high absolute correlations with S.D. These results indicate that except for *J*_g_, there is no significant singular linear relationship between the flotation variables and S.D. This relatively low correlation could be due to the inherent limitations of the statistical analysis (time-series analysis in the time domain) for differential pressure fluctuations.^18^ Pressure fluctuation is not only related to the amplitude of pressure fluctuation but also related to the frequency of different pressures.^17,26^ Thus, the application of time-series analysis in the frequency domain (spectral analysis) and state space (chaos analysis) will be employed to improve the prediction accuracy of gas dispersion of the collection zone in a column flotation based on pressure fluctuations. Thus, for assessing the hydrodynamic conditions by S.D., nonlinear modeling also should be considered.

**Table 1:** Pearson Correlations between S.D. and the Examined Flotation Variables

variables	*n*	*C*_f_	*J*_w_	*J*_g_
S.D.	–0.34	–0.26	–0.03	0.65

### 2.2 Hydrodynamic Assessment

**Figure 8:**
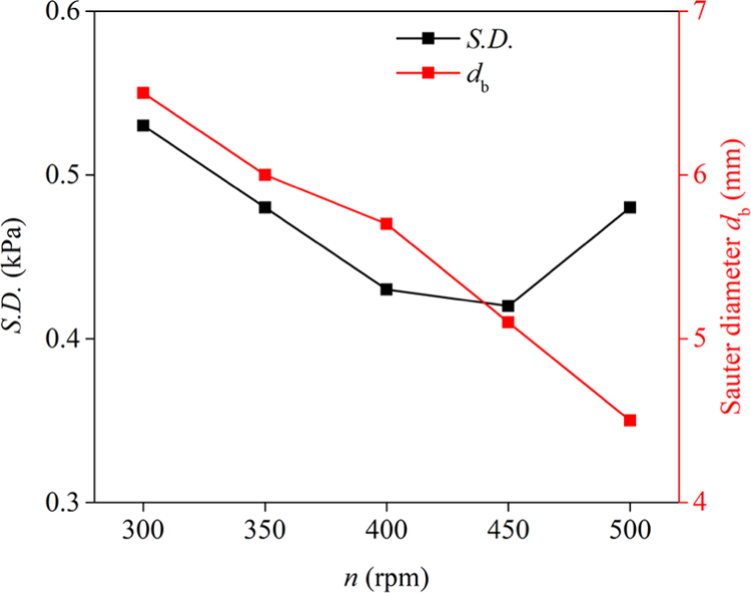
Effect of the pump speed (*n*) on the S.D. of differential pressure fluctuations and Sauter bubble diameter (*C*_f_ = 0.15 mmol/L, *J*_w_ = 0.043 cm/s, *J*_g_ = 1.44 cm/s).

**Figure 9:**
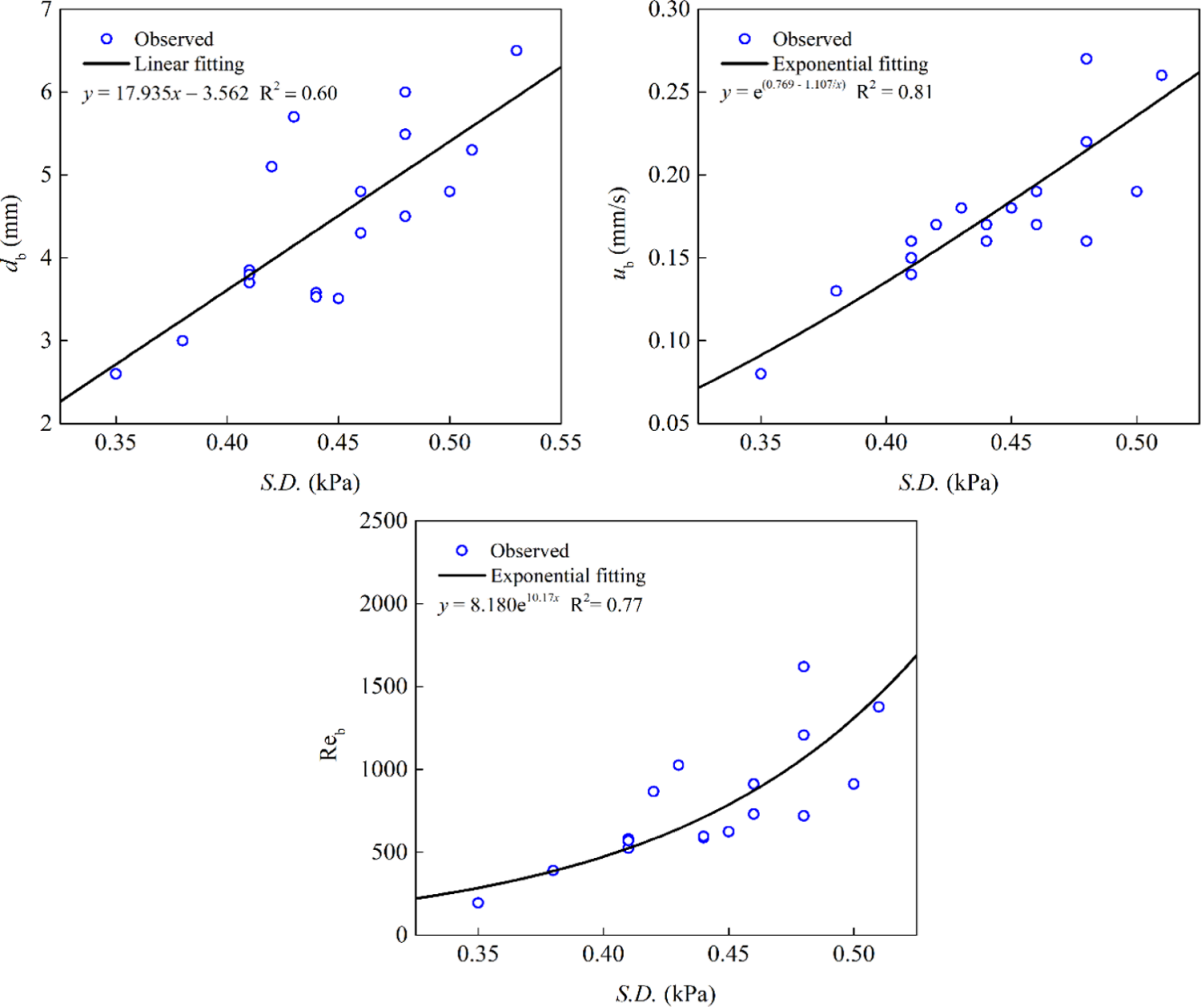
Prediction of hydrodynamics parameters based on the S.D. of differential pressure fluctuations.

The Pearson correlation assessments showed strong linear correlations (Table 2) between the differential pressure fluctuations and the collection zone’s hydrodynamic variables. The experimental and calculated data for all conditions can be found in the Supporting Information. The |*r*| values between the hydrodynamic variables were higher than those for the other flotation parameters, indicating the significant capability of S.D. to predict hydrodynamic variables. The results indicated that the S.D. of differential pressure fluctuations could accurately predict the hydrodynamic parameters (Figure 8). This may be due to the direct relationship between the average bubble velocity and the amplitude of fluctuating pressure. The Reynolds number of the bubble is related to the bubble size, bubble velocity, liquid density, and liquid viscosity, which is a determining factor for bubble–particle collision, attachment, and detachment processes.^39^ However, the bubble velocity and bubble diameter are difficult to measure in industrial flotation columns. Therefore, the measurement of pressure fluctuations and its time-series analysis can provide an alternative method for predicting gas dispersion (or hydrodynamics) in a flotation column. Notably, it is still challenging to extend the S.D.’s pressure for industrial columns. However, the S.D.’s pressure gives a possible method to measure the hydrodynamics of a flotation column. The applications of the S.D.’s pressure in gas–liquid–solid bubble column reactors have provided reference to our future research. More accurate analysis methods, including frequency domain and state space, will provide more information (Figure 9).

**Table 2:** Pearson Correlations between the S.D. of Differential Pressure Fluctuations and Hydrodynamic Parameters

variables	*d*_b_	*u*_b_	*Re*_b_
S.D.	0.77	0.87	0.82

## 3 Conclusions

In flotation columns, measuring the standard deviation of differential pressure fluctuations of the collection zone is a straightforward assessment that can be accurately performed during the process. The statistical assessment outcomes revealed that superficial gas velocity had the highest positive linear correlation with the standard deviation of differential pressure fluctuations. Pearson correlation results indicated that the relationships between the standard deviation of differential pressure fluctuations and hydrodynamic flotation variables (Reynolds number of the bubbles, bubble velocity, and bubble diameter) are higher than those of other effective flotation parameters (pump speed, superficial gas velocity, superficial water velocity, and frother dosage). The overall outcomes suggested that the standard deviation of differential pressure fluctuations is a promising tool to determine the hydrodynamic characteristics of the collection zone in flotation columns precisely (*R*^2^ > 0.77).

## 4 Experimental Setup and Procedure

### 4.1 Flotation Column Setup

**Figure 10:**
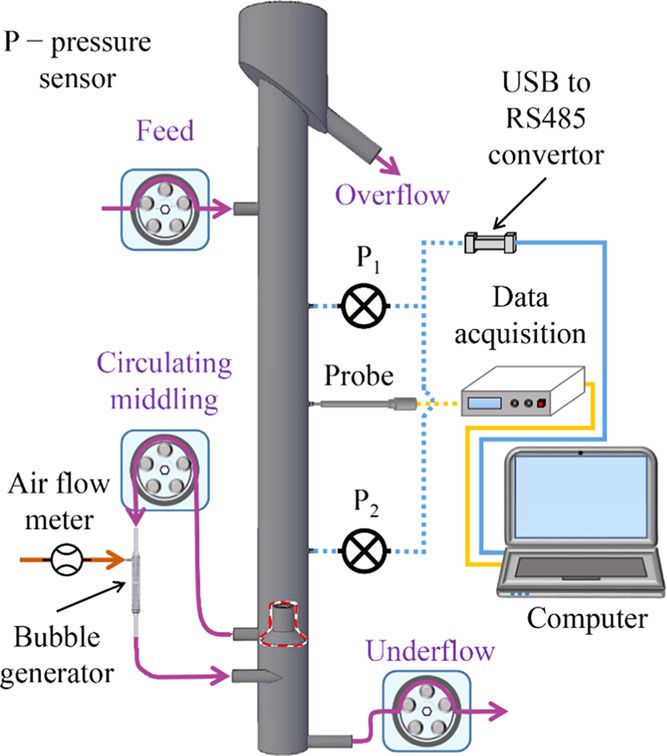
Schematic view of the experimental setup.

For the experimental setup (Figure 10), BT/301S peristaltic pumps (Lead Fluid Technologies Inc., Baoding, China) with 17^#^ hoses (6.4 mm inner diameter, 9.6 mm outer diameter) were used as the feed and underflow pumps. A TL00-700M peristaltic pump with an 82^#^ hose (12.7 mm inner diameter, 19.3 mm outer diameter) (Tianli Fluid Industrial Equipment Factory, Wuxi, China) was considered as the circulating pump. A self-aerating bubble generator was made according to the venturi tube principle and installed in the system. A cyclonic microbubble flotation column (90 mm inner diameter, 100 mm outer diameter) was constructed using acrylic glass columns. The height of the column is 1.40 m. Compared to the conventional flotation columns, this study’s flotation column has an additional zone called the cyclonic zone at the bottom.^40^ The circulating middling was extracted from the vicinity of an inverted funnel structure and then pumped into the nozzle of the bubble generator.^41^ The middling exiting the bubble generator was then tangentially fed into the cyclone section at the column’s bottom. The column was operated continuously for 3 min to reach a steady state, and then the pressure and conductance signals were collected for 10 min. *sec*-Octyl alcohol (analytical grade, Sinopharm Group Co., Ltd., Hong Kong, China) was used as a frothing reagent. In fact, the gas flow rate of the self-aerating bubble generator is directly related to the liquid recirculation rate. The circulation rate was kept constant at a relatively high pump speed of 500 rpm; thus, a sufficiently high gas flow rate can be obtained. On this basis, we control the gas flow rate using a gas flow meter. The superficial velocity of gas was calculated as *J*_g_ = *Q*_g_/*A*, where *Q*_g_ is the volumetric flow rate of the gas and *A* is the column’s cross-sectional area. The superficial water velocity represents the bias water velocity between the feed and the underflow
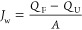
1where *Q*_F_ and *Q*_U_ are the rate of flow of the feed and underflow, respectively.

Froth depth is an important factor for the flotation column. However, it is impossible to adjust the froth depth with a constant superficial gas velocity, superficial water velocity, frother dosage, and circulating pump speed. Thus, for the gas–liquid flow, the main attention of this study is paid to the variations in the gas dispersion of the collection zone at different variables (superficial gas velocity, superficial water velocity, frother dosage, and circulating pump speed). The column was operated continuously to reach a steady state over 3 min, which was confirmed by the constant tailings and froth flow. After that, the measurements of pressure signals and gas dispersion parameters are performed.

### 4.2 Pressure signal measurement

Pressure sensors (CYYZ11) were prepared from Beijing Star Sensor Technology Co., Ltd. The measurements by pressure sensors 1 and 2 were performed 400 and 900 mm above the flotation column’s bottom, respectively. A USB converted the pressure signal to an RS485 convertor (UT-885, UTEK Technology (Shenzhen) Co., Ltd.). Notably, there is a significant difference in the frequency zones between true pressure fluctuations (resulting from bubble coalescence, bubble rising behavior, bubble breakup, etc.). The noise signals resulted from the running pumps and the environment, as reported by other investigations.^17,26^ Thus, a filter module was used to get the final pressure signal. A program written using the software package MATLAB was used for data acquisition and storage at a frequency of 250 Hz. The standard deviation (S.D.) of differential pressure fluctuations (Δ*P*) between pressure sensors 1 and 2 was calculated as
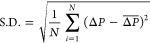
2where *N* is the total number of data points in the pressure signal and  represents the Δ*P* average value.

### 4.3 Gas Dispersion Parameters

The chord length distribution of bubbles (*d*_b_—average bubble diameter) and the average bubble velocity (*u*_b_—average bubble velocity) of the collection zone were measured by a BVW-2 multichannel bubble apparatus (Nanjing Jiuzhang Chemical Technology Co., Ltd.). The distance between the probe tip and the overflow port was 700 mm. The differential pressure was considered for the assessment since the measurement point is between the probe’s two pressure points in the BVW-2 multichannel bubble apparatus. The appearance of large bubbles is a sign of fluid transition from homogeneous flow to heterogeneous flow.^42^ Therefore, the monitoring of large bubbles can well reflect the hydrodynamic conditions.^43,44^ It is well known that many large bubbles can be used to detect regime transition from homogeneous to heterogeneous flow, and the investigation of the transition regime is quite important.^42^ Thus, the behaviors of large bubbles have a direct relationship with pressure fluctuations.

**Figure 11:**
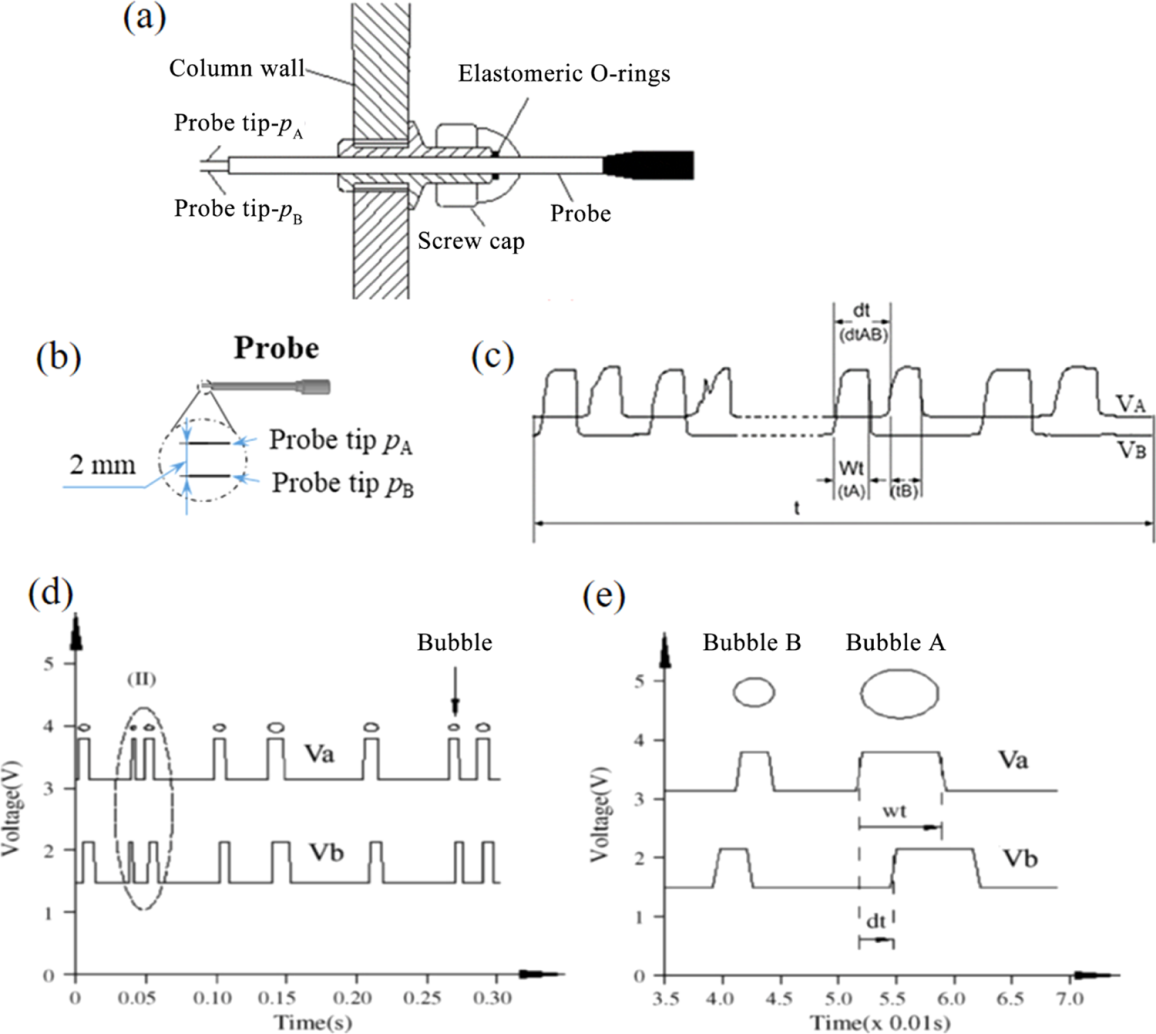
(a) Schematic diagram of the probe, (b) schematic diagram of the conductivity probe of the BVW-2 multichannel bubble parameters apparatus, and (c) processed signals from the probe tips. (d) Typical binarized signal from the conductivity probe and (e) locally enlarged signal of (II) (adapted in part from Zhang et al.^46^).

The measurement principle was based on the local conductivity in the flotation column by using two probes arranged at a certain distance (Figure 11a). The apparatus principle is based on the variations in the medium conductivity at the sensitive tip. The vertical distance between the two tips of the apparatus is 2 mm (Figure 11b). The probe was placed at a height of 700 mm above the flotation column bottom. For ensuring that the two probe tips A and B puncture the same bubble, they are placed horizontally inside the flow. The AC voltage signal (generated by the signal excitation source) was applied for the probe. When the bubble moves upward, probe tips “*p*_A_ and *p*_B_” successively puncture the same bubble, and the conductance value at the tip of the probe changes. After the detection, amplification, level adjustment, conversion, and other circuits, the probe tips’ processed voltage signals were formed and recorded by the computer (Figure 11c). These two sensitive tips (*p*_A_ and *p*_B_) were aligned with the bubble rise direction; thus, the rising bubbles could be detected sequentially. These processes would be based on the conductivity variation originated from two-level voltages, “Va and Vb”, after the level splitter. The signals were sampled, recorded, and binarized.^44^ The trigger level was chosen to avoid noises, which is 10% of the number of bubbles that their typical passage rise signals.^32^Figure 11d shows a general binarized signal that could be obtained from the conductivity probe and was converted to the local enlarged typical binarized signal to ensure probing the same bubbles through the process.^43^ Thus, according to the significant difference of conductivity between two-phase gas and liquid, the conductivity probe can accurately reflect the gas phase (bubble), change the process at its location, and convert it into a corresponding electrical signal. Then, the bubble velocity distribution and bubble size distribution could be calculated. In this study, the measured chord length distribution and average bubble velocity represent larger bubbles (larger than the distance between the two probes).^45^

The BVW-2 analysis software provided the measurement results of the chord length distribution and average bubble velocity. The frequency of collecting data with a conductivity probe is 10 kHz, and each experimental point collects no less than 1000 bubbles’ data. The bubble velocity and size for a bubble were calculated using eqs 3 and 4, respectively. The *u*_b_ values used in this study were calculated by the weighted average method via the measurement results of 1000 bubbles. The *d*_b_ value is the Sauter diameter.

The bubble velocity is calculated as
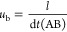
3where *l* is the distance between probe tips A and B; d*t*(AB) is the bubble time lag in the signals from the two sensing tips, i.e., the time difference between the bubble encountering the first tip and the second tip (Figure 11b). The bubble size is calculated as
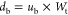
4where *W*_t_ is the duration time of the signals (Figure 11b). The Reynolds number of the bubbles (*Re*_b_) is calculated as^47^
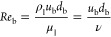
5where *ν* is the kinematic viscosity of water at 20 °C (1.0035 × 10^–6^ m^2^/s).

## Supporting Information Available

Experimental and calculated data for all conditions (PDF)
