# Supplementary material for: Exploring the Relationships between Gas Dispersion Parameters and Differential Pressure Fluctuations in a Column Flotation
Source: ACS Omega. 2021 Aug 16;6(34):21900–8. doi: 10.1021/acsomega.1c01955 (PMC8412904; doi:10.1021/acsomega.1c01955)
Supplement: Supplementary file 1 — ao1c01955_si_001.pdf [file ao1c01955_si_001.pdf]

Exploring relationships between gas dispersion parameters and differential pressure fluctuations in a column flotation

Xiangning Bu[1,*], Shaoqi Zhou[1], Meng Sun[2], Muidh Alheshibri[3,4], Md. ShakhaoathKhan[5], Guangyuan Xie[1], Saeed Chehreh Chelgani[6,*]

[1]Key Laboratory of Coal Processing and Efficient Utilization of Ministry of Education, School of Chemical Engineering and Technology, China University of Mining and Technology, Xuzhou, Jiangsu 221116, China;

[2] Fengxian Power Supply Co., Ltd., State Grid Jiangsu Electric Power Co., Ltd., Fengxian, Jiangsu 221700, China;

[3]Department of Basic Science, Deanship of Preparatory Year and Supporting Studies, Imam Abdulrahman Bin Faisal University, P.O. Box 1982, Dammam 31441, Saudi Arabia;

[4]Basic & Applied Scientific Research Center, Imam Abdulrahman Bin Faisal University, P.O. Box 1982, Dammam 31441, Saudi Arabia.

[5]ARC Research Hub for Computational Particle Technology, Department of Chemical Engineering, Monash University, Clayton, VIC, 3800, Australia

[6]Minerals and Metallurgical Engineering, Dept. of Civil, Environmental and Natural Resources Engineering, Luleå University of Technology, SE-971 87 Luleå, Sweden

□Corresponding authors:

E-mail addresses: xiangning.bu@foxmail.com, xiangning.bu@cumt.edu.cn (X., Bu);

saeed.chelgani@ltu.se (S. Chehreh Chelgani)

Table S1. The experimental results for all conditions.

Frother dosage (Cf)(mmol/L)	Superficial water flow velocity (Jw)(cm/s)	Superficial gas velocity (Jg)(cm/s)	Circulating pump speed (n) (rpm)	S.D. values of pressure fluctuations (kPa)	Sauter bubble diameter (mm)	Average bubble velocity (ub) (cm/s)	Reynolds number of the bubbles Reb (-)	Bubble surface area flux S_b (s^{-1})
0.15	0.043	1.83	500	0.51	5.3	0.26	1378	20.7
0.15	0.043	1.7	500	0.46	4.8	0.19	912	21.3
0.15	0.043	1.44	500	0.41	3.8	0.15	570	22.7
0.15	0.043	1.18	500	0.38	3	0.13	390	23.6
0.15	0.043	0.88	500	0.35	2.6	0.08	195	20.3
0.15	0.043	1.44	500	0.41	3.8	0.15	570	22.7
0.15	0.096	1.44	500	0.41	3.7	0.16	581	23.4
0.15	0.134	1.44	500	0.44	3.58	0.16	587	24.1
0.15	0.182	1.44	500	0.44	3.53	0.17	596	24.5
0.15	0.22	1.44	500	0.45	3.51	0.18	625	24.6
0.15	0.043	1.44	300	0.53	6.5	0.32	2080	13.3
0.15	0.043	1.44	350	0.48	6	0.27	1620	14.4
0.15	0.043	1.44	400	0.43	5.7	0.18	1026	15.2
0.15	0.043	1.44	450	0.42	5.1	0.17	867	16.9
0.15	0.043	1.44	500	0.48	4.5	0.16	720	19.2
0.15	0.043	1.44	550	0.5	4.8	0.19	912	18.0
0.23	0.043	1.44	500	0.41	3.85	0.14	524	22.4
0.15	0.043	1.44	500	0.41	3.8	0.15	570	22.7
0.08	0.043	1.44	500	0.46	4.3	0.17	731	20.1
0.04	0.043	1.44	500	0.48	5.49	0.22	1208	15.7
